# Supplementary material for: CT texture analysis of tonsil cancer: Discrimination from normal palatine tonsils
Source: PLoS One. 2021 Aug 11;16(8):e0255835. doi: 10.1371/journal.pone.0255835 (PMC8357133; doi:10.1371/journal.pone.0255835)
Supplement: S1 Table — (PDF) [file pone.0255835.s001.pdf]

S1 Table. Results of correlation between texture analysis and ^{18}F-FDG PET/CT parameters

		SSF0		SSF2		SSF3		SSF4		SSF5		SSF6	
		Pearson	p value	Pearson	p value	Pearson	p value	Pearson	p value	Pearson	p value	Pearson	p value
SUVmax	Mean	0.28	0.04*	-0.19	0.17	-0.22	0.1	-0.21	0.13	-0.18	0.2	-0.13	0.33
	SD	0.004	0.98	0.15	0.28	0.14	0.3	0.1	0.47	0.05	0.75	-0.02	0.86
	Entropy	0.03	0.83	0.17	0.21	0.18	0.19	0.13	0.33	0.07	0.61	0.003	0.98
	MPP	0.27	0.048*	0.06	0.66	0.03	0.85	-0.03	0.86	-0.07	0.62	-0.1	0.47
	Skewness	-0.03	0.81	0.1	0.48	0.12	0.38	0.15	0.29	0.2	0.16	0.24	0.08
	Kurtosis	0.09	0.5	-0.08	0.57	-0.11	0.44	-0.05	0.74	0.04	0.8	0.08	0.56
SUVmean	Mean	0.3	0.03*	-0.19	0.18	-0.21	0.12	-0.2	0.15	-0.17	0.22	-0.13	0.36
	SD	0.03	0.81	0.2	0.15	0.17	0.23	0.11	0.41	0.06	0.68	-0.02	0.9
	Entropy	0.07	0.61	0.22	0.11	0.2	0.14	0.15	0.29	0.08	0.58	-0.006	0.96
	MPP	0.29	0.03*	0.13	0.37	0.06	0.69	-0.01	0.95	-0.05	0.71	-0.09	0.53
	Skewness	0.02	0.88	0.18	0.21	0.17	0.22	0.19	0.16	0.24	0.09	0.24	0.08
	Kurtosis	0.02	0.91	-0.13	0.35	-0.17	0.23	-0.09	0.53	-0.01	0.96	0.04	0.79
TLG	Mean	0.14	0.33	-0.34	0.01*	-0.35	0.01*	-0.34	0.01*	-0.31	0.02*	-0.26	0.06
	SD	-0.04	0.76	-0.074	0.6	0.01	0.92	0.04	0.8	0.02	0.91	-0.01	0.95
	Entropy	-0.03	0.86	-0.04	0.77	0.05	0.7	0.09	0.52	0.08	0.57	0.06	0.67
	MPP	0.12	0.4	-0.16	0.25	-0.12	0.4	-0.11	0.42	-0.14	0.31	-0.16	0.25
	Skewness	0.17	0.23	0.16	0.24	0.18	0.2	0.16	0.24	0.2	0.14	0.28	0.04*
	Kurtosis	0.09	0.52	0.13	0.35	0.11	0.43	0.08	0.57	0.12	0.38	0.19	0.16
SUV max T/N	Mean	0.21	0.13	-0.11	0.44	-0.14	0.30	-0.15	0.29	-0.14	0.31	-0.13	0.36
	SD	0.18	0.2	0.25	0.07	0.22	0.11	0.19	0.18	0.15	0.27	0.11	0.43
	Entropy	0.2	0.14	0.27	0.04*	0.25	0.07	0.21	0.13	0.17	0.21	0.14	0.31
	MPP	0.21	0.12	0.17	0.23	0.11	0.43	0.05	0.70	0.01	0.94	-0.02	0.91
	Skewness	-0.11	0.44	0.02	0.9	0.01	0.95	0.03	0.84	0.09	0.53	0.17	0.21

	Kurtosis	0.03	0.82	-0.13	0.34	-0.17	0.23	-0.14	0.30	-0.06	0.66	-0.01	0.95
SUVmean T/N	Mean	0.20	0.14	-0.04	0.76	-0.06	0.67	-0.07	0.63	-0.07	0.61	-0.07	0.62
	SD	0.28	0.04*	0.36	0.01*	0.32	0.02*	0.29	0.03*	0.26	0.06	0.21	0.13
	Entropy	0.30	0.03*	0.36	0.01*	0.33	0.02*	0.30	0.03*	0.26	0.06	0.22	0.11
	MPP	0.22	0.12	0.28	0.04*	0.21	0.13	0.16	0.26	0.12	0.39	0.08	0.54
	Skewness	-0.11	0.43	0.02	0.88	-0.03	0.85	0.00	1.00	0.09	0.52	0.18	0.19
	Kurtosis	-0.03	0.81	-0.18	0.19	-0.20	0.15	-0.15	0.27	-0.09	0.52	-0.04	0.75
TLG T/N	Mean	0.29	0.03*	-0.18	0.19	-0.18	0.20	-0.15	0.29	-0.11	0.42	-0.07	0.62
	SD	0.14	0.31	0.10	0.46	0.14	0.31	0.16	0.24	0.17	0.21	0.17	0.22
	Entropy	0.15	0.28	0.14	0.32	0.17	0.21	0.19	0.16	0.20	0.15	0.20	0.14
	MPP	.300*	0.03	0.02	0.88	0.02	0.89	0.02	0.86	0.02	0.89	0.03	0.85
	Skewness	-0.07	0.59	0.02	0.89	-0.02	0.86	-0.06	0.66	-0.05	0.69	-0.01	0.96
	Kurtosis	0.05	0.71	-0.01	0.94	-0.02	0.91	0.01	0.92	0.11	0.44	0.18	0.20

* Statistically significant

TLG total lesion glycolysis, T/N tumor to normal ratio
