# Supplementary material for: CT texture analysis of tonsil cancer: Discrimination from normal palatine tonsils
Source: PLoS One. 2021 Aug 11;16(8):e0255835. doi: 10.1371/journal.pone.0255835 (PMC8357133; doi:10.1371/journal.pone.0255835)
Supplement: Attachment — Submitted filename: Response to Reviewers.docx [file pone.0255835.s002.docx]

**Point by point responses.**

Thank the editor and reviewers for their very thoughtful suggestions and comments that were very helpful for revising the manuscript. Followings are descriptions on the point-by-point corrections made in the revised manuscript and answers to the questions raised by the reviewers.

**Reviewer(s)' Comments to Author:**

**

Reviewer #1: comments:

· The statistical analysis in this paper is suitable.

· In terms of experimental technique, this paper is novel

**Response:** Thanks for the kind review and words.

· I would like to see some discussion of the findings of the papers with existing values (entropy) in other papers

**Response:** in response to the comment, one more reference was added as follows: Meyer et al. reported that CT entropy was correlated with hypoxic related pathological parameter, hypoxia-inducible factor-1-alpha expression.

Minor comments:

· In several places, you've used the term “we”, but it seems unnecessary.

**Response:** in response to the comment, we corrected most of sentences.

· The author can add Block Diagram of the Work.

**Response:** We added as table 1 as follows.

If the above changes made in manuscript, I recommend to publish the same
